# Antibacterial Layer-by-Layer Coatings for Medical Implants

**DOI:** 10.3390/pharmaceutics13010016

**Published:** 2020-12-24

**Authors:** Ane Escobar, Nicolas Muzzio, Sergio Enrique Moya

**Affiliations:** 1 Center for Cooperative Research in Biomaterials (CIC biomaGUNE), Basque Research and Technology Alliance (BRTA), Paseo de Miramon 182 C, 20014 Donostia-San Sebastian, Spain; ane.escobar@i3bs.uminho.pt; 2 Department of Biomedical Engineering and Chemical Engineering, University of Texas at San Antonio, San Antonio, TX 78249, USA; nicolas.muzzio@utsa.edu

**Keywords:** bacterial infections, polyelectrolytes, Layer-by-Layer, antibacterial coatings, antiadhesive surfaces, localized action, medical implants

## Abstract

The widespread occurrence of nosocomial infections and the emergence of new bacterial strands calls for the development of antibacterial coatings with localized antibacterial action that are capable of facing the challenges posed by increasing bacterial resistance to antibiotics. The Layer-by-Layer (LbL) technique, based on the alternating assembly of oppositely charged polyelectrolytes, can be applied for the non-covalent modification of multiple substrates, including medical implants. Polyelectrolyte multilayers fabricated by the LbL technique have been extensively researched for the development of antibacterial coatings as they can be loaded with antibiotics, antibacterial peptides, nanoparticles with bactericide action, in addition to being capable of restricting adhesion of bacteria to surfaces. In this review, the different approaches that apply LbL for antibacterial coatings, emphasizing those that can be applied for implant modification are presented.

## 1. Introduction

A current challenge in the design of biomaterials for tissue engineering and implant fabrication is the need to inhibit bacterial adhesion/colonization while, at the same time, facilitating the desired interactions of the implant with cells and proteins [1]. Depending on the desired application one may aim to promote cell adhesion, migration, proliferation and differentiation, as in the case of tissue regeneration therapies [2,3], or prevent it, as in some surgical materials, or in biomaterials to prevent post-operative adhesions [4,5].

The widespread occurrence of bacterial infections and their increasing resistance to antibiotics has led to the development of a wide variety of antibacterial coatings for multiple medical settings, especially bone implants [6]. The surface of an implant can be susceptible to numerous bacterial infections largely due to the formation of a biofilm on its surface, which triggers a compromised immune response at the implant/tissue interface [7]. Once a biofilm is formed, it protects adherent bacteria from the host defence system and bactericidal agents via several mechanisms [8,9,10]. The biofilm becomes a source of pathogens and infections, being the cause of so-called nosocomial infections [11,12,13]. Because biofilms can develop on almost any material present in a surgical theatre, preventing their formation is fundamental for patient survival.

Even with the development of therapies based on prophylactic antibiotics, environmental control and the latest improvements in surgical techniques, the incidence of infection varies between 1–2% in primary arthroplasties and 3.5–5% in revision surgeries. [14,15]. For example, the main risk factors associated with infection in knee arthroplasty are a previous surgery, advanced age, female gender, rheumatoid arthritis, obesity, diabetes and immunodeficiency diseases [16]. Post-implantation surgical trauma weakens the local defence system, raising the risk of infection, which if prolonged requires further surgical interventions to replace the implant [17]. The revision surgery, however, is more challenging than the initial surgery because of higher risk of trauma and the need of a longer period for bone healing [18]. Once the implant is in contact with physiological media, a protein layer is formed on the surface, facilitating bacterial colonization and in the worst case scenario, biofilm formation [8,19]. Biofilms act as a protecting layer against the host defence and bactericidal agents, so it remains highly challenging and critical to avoid infection just after the implantation surgery [8].

If there is still no need to replace the implant, infection focus has to be removed surgically, which is followed by a long time treatment with antibiotics [13]. Two-thirds of the infections in orthopaedic implants are caused by *Staphylococcus* pathogens. These also are the responsible for two of the major bone infections: arthritis and osteomyelitis, which suppose the inflammatory destruction of joints and bones [20]. More specifically *Staphylococcus aureus* and *Staphylococcus epidermidis* account together for two out of three infections in orthopaedics [20].

In the last few decades several approaches have been developed for bringing antibacterial functions to the implants as a more effective alternative to systemic delivery of antibiotics [21]. The different approaches developed aim to preserve antibacterial activity through the process of wound healing and capsulate formation [21]. Despite the highest risk of bacterial infections is during operation when the body is open to the air bacterial infections can take place at any time before capsule closing [22]. By making the surface of the implants antibacterial undesired effects of delivering large doses of antibiotics systemically or at the site of implant could be avoided [22]. Many of the strategies developed for bringing antibacterial properties to implants are based on their functionalization with polymer films, which either act themselves as antibacterial or are used to encapsulate antibiotics, bring antibacterial functions or decrease bacteria adhesion on the implant [23,24].

In this review a focus on one type of polymeric coating: polyelectrolyte multilayers (PEMs) fabricated by the Layer-by-Layer (LbL) technique is provided. PEMs are prepared by the sequential assembly of oppositely charged polyelectrolytes. The technique can be applied for functionalization of any charged surface requiring simple and inexpensive procedures. PEM fabrication offers a modular approach to bring antibiotics, bactericide peptides and nanoparticles, positive charges to disrupt bacteria membranes, and inhibit bacteria adhesion and proliferation, all in one [25].

## 2. Nosocomial Infections

The term nosocomial infections refers to infections associated with healthcare and acquired in a hospital [26]. These are infections secondary to the main condition of the patient, and can have lethal consequences following operations such as bone replacement or open heart surgery [27,28,29]. *Staphylococcus aureus* is an important nosocomial pathogen, able to cause a variety of conditions. It can often be found as a commensal and a transient, or persistent part of the resident flora of the skin and anterior nares in a large proportion (20–50%) of the human population. However, when cutaneous/mucous barriers are breached, severe and at times life-threatening infections can develop. Nosocomial infections of *S. aureus* are particularly frequent in immunocompromised and severely debilitated patients and prevail in the presence of indwelling medical devices. The treatment of *S. aureus* infections is often complex, namely due to the emergence of methicillin-resistant *S. aureus* (MRSA) strains and resistance to other classes of antibiotics. Because of its pathogenic potential and the complexity of its treatment, MRSA has received more attention than its methicillin-sensitive counterpart (MSSA). MRSAs are resistant to β-lactam antibiotics (e.g., oxacillin, penicillin and amoxicillin), including third generation cephalosporins, streptomycin, tetracycline and sulphonamides; and upon exposure to vancomycin and other glycopeptide antibiotics, certain MRSA strains become less susceptible to these antibiotics. *S. aureus* possesses several cell-surface adhesive molecules that facilitate its binding to the bone matrix. Binding involves a family of adhesins that interact with the extracellular matrix (ECM) components, and these adhesins have been termed microbial surface components recognizing adhesive matrix molecules (MSCRAMMs). Specific MSCRAMMs are needed for the colonization of individual tissues and for adhesion to biomaterials and to the ECM proteins deposited on the biomaterial surface. MSCRAMMs include fibrinogen-binding proteins, elastin-binding adhesin and collagen-binding adhesin. A number of these adhesins have already been thoroughly investigated and identified as critical virulence factors involved in various phases of infection, including early colonization, invasion, tissue localization and cell internalization [30,31,32]. In recent years, the polysaccharide intercellular adhesin (PIA) has been found in many *S. aureus* strains, and is required for biofilm formation and bacterium-bacterium adhesion [33]. This adhesin is responsible for the production of the extracellular polysaccharide matrix that develops the biofilm. It is known that once a biofilm is formed, the bacteria within the biofilm are protected from phagocytosis and antibiotics [32].

*S. aureus* produces virulence factors to facilitate disease progression, and rapidly develops antimicrobial resistance. Virulence factors include the MSCRAMMs as receptors in the human host, other surface proteins, polysaccharide intercellular adhesion, and capsular polysaccharides. The cell-surface MSCRAMMs typically are produced during the exponential growth phase. The role of these various virulence factors is to provide nutrients required for survival in the host, and microbial cell protection from the host immune system during lesion formation. The secreted virulence factors, typically produced during the post-exponential and stationary phases, include a large group of exoenzymes, such as proteases, glycerol ester hydrolase and nucleases that make nutrients available to the microorganism [32].

## 3. Coatings for Titanium Implants

The increased use of titanium and its alloys as biomaterials for bone implants in substitution/repair approaches is a result of their superior biocompatibility and excellent low corrosion characteristics [34,35]. Since the role of an implant is the replacement of the bone, it needs to mimic the biological environment and mechanical properties of the bone. Although currently used commercial titanium implants satisfy the required mechanical properties with a Young’s modulus within the range of 100 and 120 GPa [36], the chemical instability and deformations of the protective oxide layer usually results in poor osteointegration. Surface deformations of the protective layer liberate metal ions which form Lewis acids and lower the pH in the implant environment [37]. The acidic environment formed at the implant surface favours bacterial infection and causes an immunogenic response which can lead to an aseptic loosening of the implant and a second surgery is needed to replace the infected implant [17,38].

It is well known that the physicochemical properties of the surface/material such as surface charge, topography, hydrophilicity/hydrophobicity, stiffness, etc., can selectively promote or prevent the absorption of proteins, and the adhesion of cells and bacteria [39,40]. A large body of work has been dedicated to the development of proper coatings for titanium implants, which make the surface antibacterial and resistant to bacterial adherence [41]. Due to the diversity of bacterial ecosystems, the coatings should be tailored to tackle specific bacteria strains. Although post-implementation supply of antibiotics to the patients can prevent infection, the low drug concentration reaching the target site remains a major disadvantage [17].

The encapsulation of antibiotics in polymer matrices is an attractive approach for the fabrication of antibacterial coatings. Polymer coatings can be made out of hydrogels, Layer-by-Layer (LbL) assemblies, polymer brushes, or porous polymer scaffolds [1,42,43,44,45,46,47,48,49,50,51,52]. A major drawback of the encapsulation of antibiotics in polymer films is the difficulty in achieving a slow release of antibiotics. In addition, often the coating degrades accompanied by liberation of the antibiotics all at once, or at a rate faster than required [6,53]. Gentamicin, cephalothin, amoxicillin, tobramycin, or vancomycin are some of the most used antibiotics due to their broad antibacterial spectra [54,55,56,57,58].

Non-antibiotic organic antimicrobial agents have also been proposed to avoid the risk of drug resistance associated with antibiotics [17,59,60]. Inorganic materials such as silver have also been proposed as an attractive dopant for titanium implants since it exhibits a broad antibacterial spectrum. However, silver compromises the activity of osteoblasts and epithelial cells [61,62]. The treatment of the implants with UV irradiation, or by changing the surface crystallinity, leads usually to alteration of the physicochemical properties, and decreases bacterial activity [63]. Interestingly, the use of nitric oxide has also been proposed as it inhibits the growth of a wide variety of bacteria [64]. Nitric oxide augments the antimicrobial ability of the immune system, thus it is loaded into xerogels for subsequent release [65]. Nonetheless, the combination of nitric oxide with titanium implants remains unproven. Bioactive polymers coatings, such as chitosan are also investigated because they can reduce bacterial adhesion [66].

## 4. Layer-by-Layer Technique

**Figure 1:**
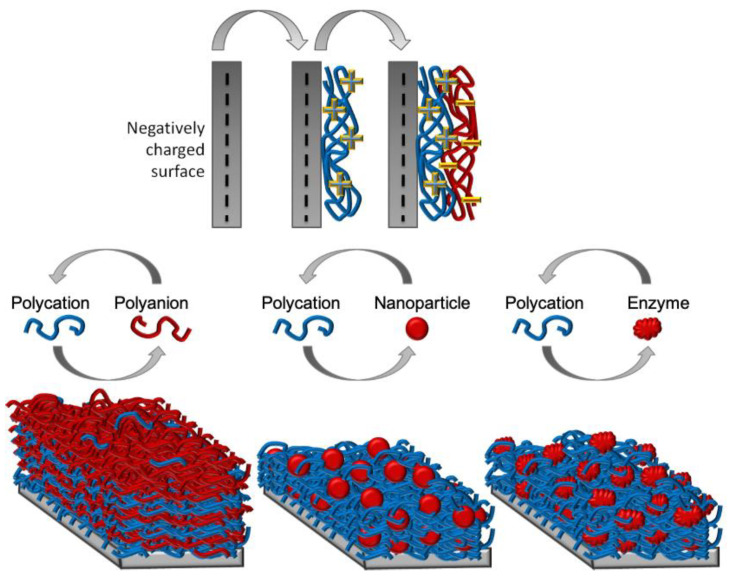
Layer-by-layer assembly method. Alternatingly, positively and negatively charged molecules are adsorbed one after the other on a substrate. This process can be repeated n times to a final film thickness. Adapted with permission from [77], Springer Nature, 2009.

By taking advantage of the large tunability offered by the LbL assembly technique and the large variety of available building blocks, several strategies have been developed based on LbL to prevent bacterial adhesion and biofilm formation. The fabrication of multilayer films of polyelectrolytes by means of the LbL technique was first shown by Decher et al. [67]. The LbL technique is based on the alternating deposition of oppositely charged polyelectrolytes on top of charged surfaces, this process is shown in Figure 1 [68,69,70]. The assembly is driven by attractive electrostatic interactions between polycations and polyanions, and entropy considerations, related to the release of counterions during polyelectrolyte assembly [71,72]. This technique is a powerful strategy for non-covalent modification of charged surfaces. LbL is an easy and reliable method for surface engineering and has many potential applications in diverse areas such as optoelectronics, nanofiltration, catalysis, and as anti-corrosion coatings [69,73,74,75]. PEMs have also found a variety of biological and biomedical applications. These include coatings to either promote or prevent cell adhesion, drug encapsulation, and directing or maintaining cellular phenotypes [76].

Initially developed for synthetic polyelectrolytes, the LbL technique has been extended to biopolyelectrolytes and other materials, which are assembled by electrostatic interactions, by hydrogen bonding, or by ion coordination in a sequential protocol. PEMs can be produced by combining a diverse array of components. Biopolymers such as proteins and nucleic acids, lipids, enzymes, and inorganic particles have been introduced in multilayer films expanding their potential for applications in multiple areas: sensing, filtration, drug delivery or energy, to name but a few [68,78,79]. In addition, PEMs can be produced by spraying polyelectrolyte solutions covering large surfaces. Spray based LbL is conceptually as easy as dipping LbL and can be applied, for example, to cover implants with diverse geometry [80].

PEMs assembled by the LbL technique can be considered a special case of polycation/polyanion complex formation. Due to the stepwise assembly of polycations and polyanions, the film gains a layered structure. However, the layers are not fully stratified, as there is a certain degree of interdigitation among them because of the free space within a deposited polyelectrolyte layer, which is filled as the number of layers deposited increases [81]. PEMs are found to be very stable; they cannot be easily removed unless one of the components loses charge by means of a change in pH, or when a specific ion, or surfactant, interacts with the polymers weakening the electrostatic interaction between the polyelectrolytes [82,83]. Only at very high ionic strength can the films be partially erased [84].

Two main types of growth kinetics have been identified in the LbL technique: (i) linear and (ii) exponential or supralinear. In linear growth the amount of polyelectrolyte assembled per layer is constant while in the exponential or supralinear, the amount of polymer assembled increases as the number of layers assembled increases, following one of these two growth laws [85,86]. A supralinear growth mechanism has the advantage that larger amounts of material can be adsorbed in each assembly step, leading for example to an increased antibiotic loading and release, as will be described in the following section.

## 5. LbL for Antibiotic Encapsulation

The LbL technique has been used for the engineering of scaffolds and implants to assemble growth factors and other molecules that facilitate tissue regeneration or enhance cell adhesion [87,88,89,90,91,92,93].

Molecular complexes, stable colloidal aggregates of molecules bound by weak interactions, have also been employed as building blocks for the fabrication of PEMs. For example, Romero et al. [94] have shown that complexes of alginate and the antiTNF-α antibody can be assembled in LbL films, while the direct assembly of the antibody does not result in stable layers because of the weak charge of antiTNF-α. PEMs assembled through the LbL technique can retain a high drug concentration, which affords many possibilities for the release of therapeutic molecules in a localized manner [95]. Drug reservoirs can be constructed in PEMs by exploiting the characteristic increase in the amount of polymer, or polymer therapeutic complexes, deposited as the number of layers increases as associated with exponentially growing PEMs, not normally observed under linear growth [96]. Drug reservoirs can be achieved by complexing a drug with the polymers assembled by LbL through electrostatics or hydrogen bonding, or by assembling layers of the drug between the polymer layers. The exponential or supralinear growth allows for a larger concentration of drug compared with linear growth and also can be used for designing a dosage profile as the top layers will entail more drug than the inner layers [97].

**Figure 2:**
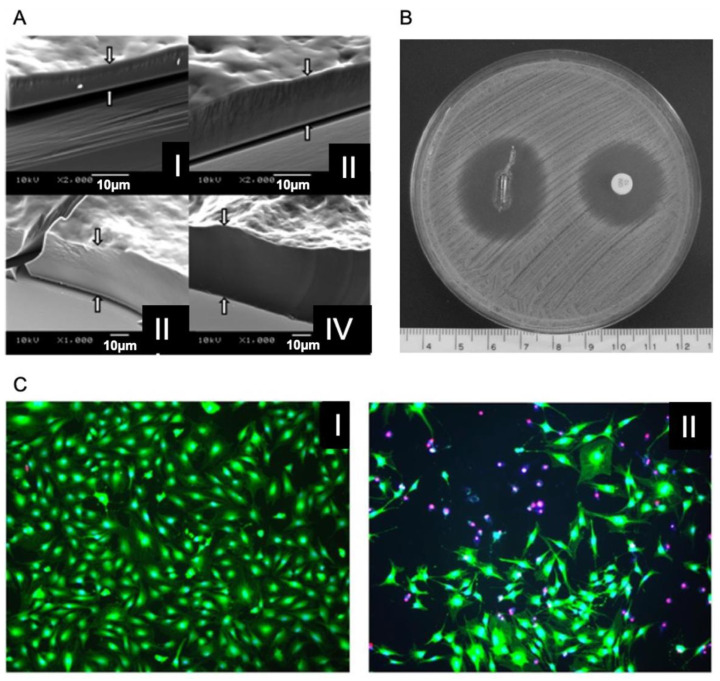
(**A**) SEM images of multilayers made of 25 (I), 50 (II), 100 (III) and 200 (IV) tetralayers. (**B**) Antibiogram; the titanium rods coated with [Poly 1/poly acrylic acid (PAA)/GS/PAA]_200_ + [Poly 1/PAA/GS]_1_ produced a baseline zone of inhibition of 25.6 mm (measured perpendicular to the long axis of the rod) against S aureus after overnight incubation at 37 °C. As a control, the sample is referenced to a commercially available BD Sensi-Disc. The lighter color at the rod surface is a result of the ruptured agar and not the presence of bacteria. The scale bar is in centimeters. (**C**) 100× images of MC3T3 cells subjected to 16–18 h treatment in (I) 100 and (II) 200 tetralayer elution buffer. Live cells are represented by a blue nucleus surrounded by green cytoplasm. Dead cells are represented by a red nucleus. Reproduced with permission from [11], Elsevier, 2010.

Moskowitz et al. [11] proposed a LbL based antibacterial coating with an initial burst release of gentamicin followed by slow release, which is essential to ensure an initial inhibition of the adhesion of bacteria during surgery, where there is the highest risk of infection as the body is open and exposed. A continued and slow release is then required to avoid bacterial infection during the formation of a protective fibrous capsule and tissue integration on the implant [98]. The coating was formed by a tetralayer unit containing gentamicin sulphate, poly acrylic acid (PAA) and a synthetic poly(β-amino ester) (Poly 1), combined as PAA/Poly 1/PAA/Gentamicin. Gentamicin is a frequently used antibiotic, an aminoglycan displaying three primary amine groups, which can interact with PAA through electrostatic interactions and hydrogen bonding. The entire film comprised up to 200 tetralayers (Figure 2A), fabricated over 5 days using an automated procedure. The coatings comprised of 100 tetralayers (Figure 2C) and had a bactericidal effect against *S. aureus* (Figure 2B), resulting in degradation products, which were generally nontoxic towards MC3T3-E1 pre-osteoblasts [11].

Albright et al. [99] developed a self-defensive multilayer using a chemically crosslinked LbL hydrogel of poly(methacrylic acid) with a covalently attached pH-sensitive SNARF-1 fluorescent layer. The coating contains gentamicin or polymyxin B. Confocal Laser-Scanning Microscopy images are reported showing that an antibiotic release is triggered by pH changes. The local acidification generated when bacteria are attached to the surface induced the release of the antibiotic, yielding a high bacterial killing efficiency for *S. aureus* and *E. coli* [99].

**Figure 3:**
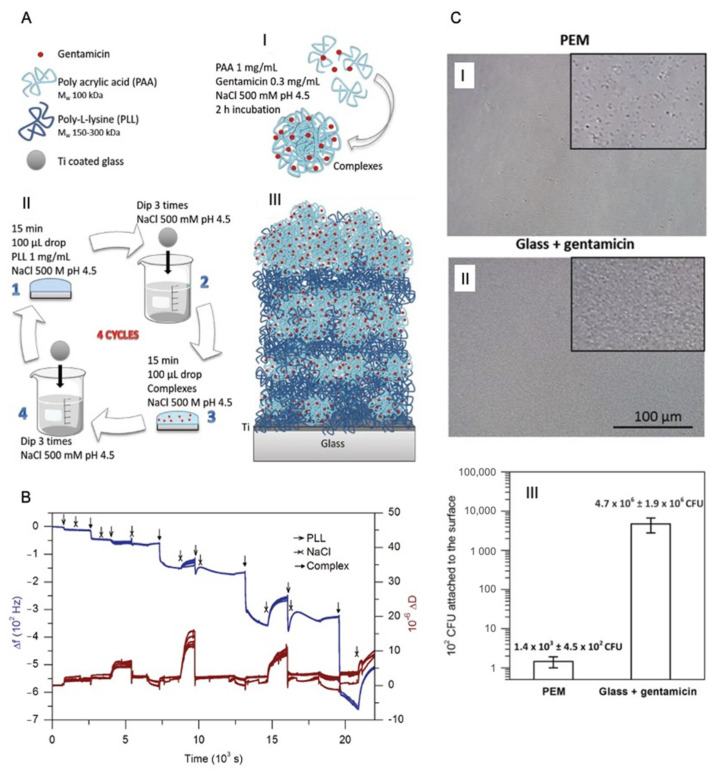
(**A**) Scheme of the formation of gentamicin and PAA complexes in 500 Å~10^−3^ m NaCl at pH 4.5 (I), LbL assembly of PLL and PAA–gentamicin complexes. The LbL assembly was performed in 500 Å~10^−3^ m NaCl at pH 4.5 in four steps: 1. 15 min incubation of 100 μL drop of 1 mg mL^−1^ PLL, 2. removal of the PLL that has not been adsorbed by dipping the substrate in 500 Å~10^−3^ m NaCl pH 4.5, 3. 15 min incubation of 100 μL drop of PAA–gentamicin complexes, and 4. removal of the complexes that have not been adsorbed by dipping the substrate in 500 Å~10^−3^ m NaCl pH 4.5. This cycle is repeated four times (II) and scheme of PEMs showing four bilayers of PLL/PAA–gentamicin complexes grown on top of titania films (III), (**B**) QCM-D monitoring of Layer-by-Layer assembly of PLL and PAA–gentamicin complexes and (**C**) I. *S. aureus* growth on top of the multilayer and II. on glass substrates immersed in gentamicin. Cell observer images following 24 h of incubation of *S. aureus* on I. top of the multilayer and II. glass immersed in gentamicin, insert images are a zoom of 4×, and III. CFU of the adhered bacteria on the PEM and glass with gentamicin. Reproduced with permission from [101], John Wiley and Sons, Inc, 2019.

De Avila et al. [100] evaluated the antibacterial efficiency of a 10 bilayer film consisting of poly(acrylic acid) (PAA) and poly-L-lysine (PLL) with tetracycline (TC) incorporated in the last layer. The multilayer is formed on top of titanium discs, and they obtained an initial burst release of TC, which has an antibacterial effect against *Porphyromonas gingivalis* [100]. A. Escobar et al. [101] prepared an antibacterial coating on titania based on the formation of complexes of PAA and gentamicin, which are assembled with poly lysine (PLL) (Figure 3A). The multilayer formation followed an exponential growth (Figure 3B) and was demonstrated to be effective in preventing the proliferation of *S. aureus* (Figure 3C) [101]. Only four layers of PAA-gentamicin complexes and PLL are needed to produce a film with a superior antibacterial performance as compared to that reported elsewhere [90] The release of gentamicin shows a two-step profile; an initial burst release followed by a prolonged release lasting more than a month. Dwivedi et al. [102] used vancomycin to decorate orthopaedic implants with antibacterial activity against *S. aureus*, without showing cytotoxic effects on L929 cells (mouse fibroblast cell line) proliferation. Vancomycin is loaded into niosomes, which are assembled in an LbL coating formulated as follows: Vancomycin/poly(lactic acid) (PLA)/niosomes, with 40 tri-layers deposited on top of the orthopaedic implant (bone plates made from stainless steel) [102]. The same research group recently reported other coatings strategies based on niosomes containing antibiotics [103,104]. They have coated dental implants alternating niosomes and PLA comprising two types of niosomes, i.e., the first ones made of sorbitan stearate (span 60) and the others of cholesterol with encapsulated minocycline [103]. The first layer is PLA, and then the niosomes are spray coated for 30, 60, 90 and 120 cycles, alternating both types of niosomes, being each cycle a deposition of span 60 and cholesterol-minocycline niosomes. Every 30 cycles the implant is dipped into the PLA solution, and then continued with the electrostatic adsorption of the alternating niosomes. As last step, 50 cycles of poly (ε-caprolactone) (PCL) nanospheres are spray coated on top of the implant. The authors observed that when PCL nanospheres are coated on top of the multilayer there is a lower initial burst release of the antibiotic. The implants in all of the formulations have shown a prolonged release of minocycline lasting for a week. The amount of minocycline released depends on the number of cycles of niosome absorption performed to coat the implant, as higher as the cycles, more antibiotic will be present on the coating. It was also demonstrated that bacterial growth of *P. gingivalis* can be inhibited and no toxic side effects have been reported [103].

Recently, Yavari et al. [105] published a work where multilayers of gelatin (Gel)/bone morphogenetic protein-2 (BMP-2) and chitosan (CHI)/vancomycin are assembled on the surface of selectively laser melted porous structures made from commercial titanium. Their objective was to simultaneously prevent implant-associated infections and stimulate bone tissue regeneration, as some other published works have previously done, [44,106] combining the use of bone-specific growth factors and antibiotics to decorate bone titanium implants [105].

## 6. LbL for Antimicrobial Peptides Encapsulation

Antimicrobial peptides (AMPs) are short peptides with a broad range of antibacterial activities [107]. They are produced by all life forms. As part of the innate immunity in higher organisms such as humans they protect them against infections. Moreover, several AMPs modulate the innate immune response of the host promoting pathogen clearance. With the global expansion of antibiotic resistance, AMPs are receiving attention as novel therapeutic agents [108,109]. In fact, it has been recently shown that antibiotic-resistant bacteria show a high frequency of collateral (increased) sensitivity to AMPs [110]. Antimicrobial peptides have been incorporated into biomaterials using different approaches such as through functionalization of thermoresponsive hydrogels [111], electrospinning [112], supramolecular interactions [113], and through the Layer-by-Layer assembly technique [114].

**Figure 4:**
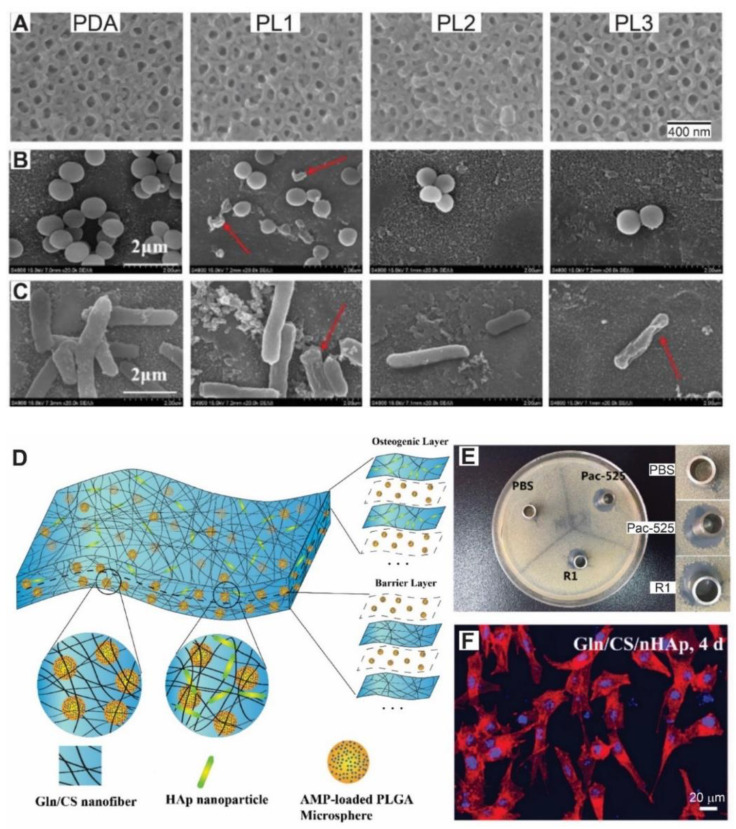
(**A**) Top view SEM images of titanium dioxide nanotubes coated with poly-dopamine (PDA), PDA grafted with on layer of ε-Polylysine (PL1), PDA coated with two layers of ε-Polylysine and one layer of gum arabic (PL2) and PDA coated with three layers of ε-Polylysine and two layer of gum arabic (PL3). Morphology of *S. aureus* (**B**) and *E. coli* (**C**) adhered to each material examined by SEM. Cracked bacteria appear on the surface (red arrows). An antiadhesive effect can be seen with the increase in the number of layers (PL2 and PL3) along with the contact-kill effect. (**D**) Schematic diagram of the fabrication and structure of the composite membrane by Layer-by-Layer electrospinning of gelatin (Gln) and chitosan (CS), and alternatively electrospraying of antimicrobial peptide (Pac-525)-loaded poly lactic-co-glycolic acid microspheres. The osteogenic layer also includes hydroxyapatite (Hap) nanoparticles. (**E**) Typical inhibition zones of *S. aureus* induced by PBS (negative control), PAC-525 (positive control) solution and the supernatant solution after one week of incubation with the composite membranes described in (**D**). (**F**) Microscopy images of rat bone marrow mesenchymal stem cells stained for actyn cytoskeleton and cell nucleus 4 days after seeding on the osteogenic layer. A, B and C adapted with permission from [115]. Elsevier, 2020. (**D**–**F**) adapted from [116], MDPI, 2018.

Zhang et al. coated an array of titanium dioxide nanotubes with a multilayer assembled with the antimicrobial peptide ε-polylysine as polication and gum Arabic (polyanion) [115]. The anodized titanium surface was initially functionalized with polydopamine in order to covalently graft the first layer of ε-polylysine. By means of SEM analysis, the authors showed that the inner diameter of the nanotubes shrank with the increase in the number of deposited layers (Figure 4A). The films presented long-term antibacterial properties up to 3 weeks against both gram-positive *S. aureus* and gram-negative *E. coli* assessed by SEM (Figure 4B,C). Those authors found cracked bacteria (bactericidal activity) and a decrease in bacteria concentration (antiadhesive effect) on multilayer-coated nanotubes. Antibacterial activity was enhanced with increasing numbers of assembled bilayers. The composite film also significantly improved the proliferation and osteogenic differentiation of rat bone marrow mesenchymal stem cells (rBMSCs) in comparison with bare polydopamine modified titanium.

Another common strategy in tissue engineering is guided tissue regeneration (GTR), i.e., a surgical procedure in which a membrane is used to assist affected tissue regeneration and as a physical barrier to generate space and prevent surrounding undesired tissue migration (epithelial and connective) into the defective area [117]. In a recent study, He et al. developed a microsphere-embedded nanofibrous membrane by sequential Layer-by-Layer electrospinning of gelatin and chitosan, and alternating electrospraying of antimicrobial peptide (Pac-525)-loaded poly lactic-co-glycolic acid microspheres [116]. The membrane comprised two blocks, the osteogenic block in which hydroxyapatite nanoparticles were electrospun along with the gelatin and chitosan, and the barrier block without these nanoparticles. A scheme of the multilayer structure and composition is depicted in Figure 4D. The membrane exhibited long-term sustained release of Pac-525, bactericidal activity for one week and bacteriostatic activity for up to a month against both *E. coli* and *S. aureus* (Figure 4E). Moreover, the osteogenic block presented good cell adhesion and proliferation and promoted osteogenic differentiation of rBMSCs. The adhesion of rBMSCs on the osteogenic block is shown in Figure 4F.

In a different approach, Xu et al. employed the Michael addition/Schiff base reaction to covalently assemble a robust and durable antibacterial multilayer on top of stainless steel [118]. The authors alternatively assembled tea stain-inspired plant polyphenolic tannic acid, which later also served as the initiator anchor, and histone-derived parasin I antifouling-antimicrobial peptide. The resulting coating exhibited good resistance to the adhesion of Gram-positive bacteria (*S. aureus* and *S. epidermidis*), Gram-negative bacteria (*Pseudomonas sp*. and *E. coli*) and microalgae (*Amphora coffeaeformis*). These antifouling properties were improved with the increase of the number of bilayers assembled. Shi et al. reported a multi-layered coating on a titanium surface with prolonged antimicrobial activity, limited biofilm formation and non-immunotoxicity for dental implant surgery applications [119]. Polished titanium plates were immersed in chitosan to provide a stable positive charge. Then, hyaluronic acid and broad-spectrum antimicrobial peptide Tet213 linked to collagen IV were assembled using the Layer-by-Layer technique. The coating yields long-term release of Tet213 linked collagen, inhibited early *S. aureus* biofilm formation and sustained growth inhibition of both Gram-negative anaerobe *P. gingivalis* and Gram-positive aerobe *S. aureus* for up to one month, which is approximately the healing period for soft tissue after implant placement. In addition, the multilayer presented good adhesion of HaCaT keratinocyte cell line, non-cytotoxicity, low erythrocyte haemolysis and had no effect on serum immunoglobulin levels. Since AMPs can be enzymatically degraded and are expensive to isolate or synthesize, Dorner et al. reported the use of polymer-based synthetic mimics of antimicrobial peptides (SMAMPs) as actively tuneable components in LbL multilayers [120]. They synthesized either hydrophobic butyl or hydrophilic diamine cationic poly(oxanorbornene)-based SMAMPs and assembled them with anionic poly(acrylic acid). By means of surface plasmon resonance spectroscopy and Zeta-potential measurements they tuned the LbL assembly conditions in order to increase film stability and optimize the antimicrobial activity, which was heavily dependent on the positive charge of the surface.

## 7. LbL for Antibacterial Nanoparticles Loading

With the increase of antibiotic resistant bacteria strands, antimicrobial metal and metal oxide nanoparticles are more often proposed as potential therapeutics and alternatives to antibiotics. Though the antibacterial mechanisms of nanoparticles are not fully understood, it is currently accepted that microbes are eliminated either by bactericidal effects such as release of free metal ions, generation of oxidative species, disruption of normal function of membranes, proteins and DNA, or by bacteriostatic effects coupled with killing by the host’s immune system [121]. It has been proposed that it is difficult for microbes to become resistant to nanoparticles, as bacteria would need multiple and simultaneous gene mutations in the same bacterial cell to develop resistance to the multiple mechanisms of action of nanoparticles [122]. Due to their effective antimicrobial properties and biocompatibility of Ag ions at low doses, Ag nanoparticles are the most promising and most explored alternative among antibacterial nanoparticles [123].

Nanoparticles as charged objects can be included in Layer-by-Layer films and deposited in between polymeric layers. LbL assembly offers a pathway to increase the amount of nanoparticles deposited on a surface, as multiple layers of nanoparticles can be assembled in a sequential fashion.

**Figure 5:**
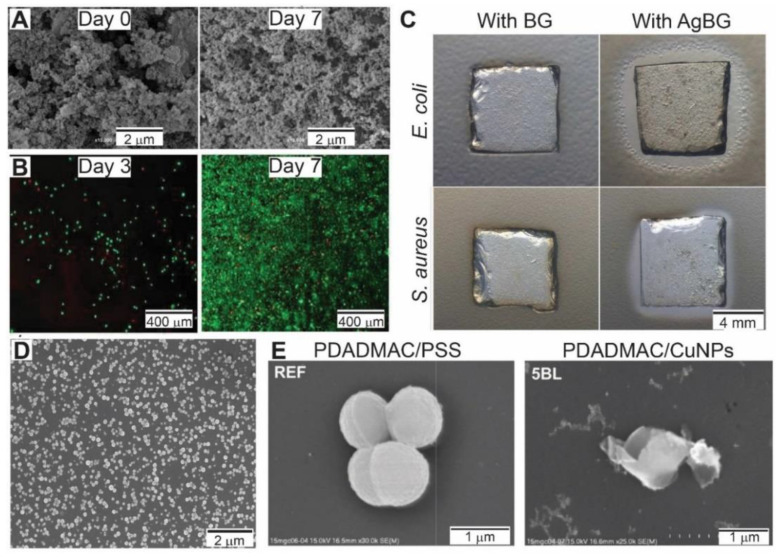
(**A**) SEM images of chitosan (CHI), dopamine-modified hyaluronic acid (HA-DN) and silver doped bioactive glass nanoparticles (AgBG) multilayer film before and after immersion on simulated body fluid solution for 7 days. (**B**) Live-dead images of L929 fibroblast cells on CHI/HA-DN/AgBG multilayers 3 and 7 days after seeding. Live cells were labelled with green and the dead cells with red. (**C**) Images of the disk diffusion methodology applied to evaluate the antimicrobial properties against *E. coli* and *S. aureus* of CHI/HA-DN multilayers with bioactive glass nanoparticles (BG) and AgBG. The antimicrobial behaviour of the film with AgBG is demonstrated by the formation of an inhibitory zone surrounding it. (**D**) SEM image of a five bilayer-thick film composed of PDADMAC and CuNPs. (**E**) SEM images of *S. aureus* on PDADMAC/PSS reference sample and 5-bilayers CuNPs-containing coatings. Typical round *S. aureus* cells forming clusters are observed on the reference sample, whereas single wrinkled bacteria with disrupted cell membrane are observed on CuNPs-containing coatings. (**A**–**C**) adapted with permission from [124], Royal Society of Chemistry, 2016. (**D**,**E**) adapted with permission from [125], Elsevier, 2019.

In reference [124] Carvalho et al. developed multifunctional mussel inspired antibacterial bio adhesive films for orthopaedic applications. By means of the Layer-by-Layer technique the authors combined positively charged polysaccharide chitosan, dopamine-modified negatively charged polysaccharide hyaluronic acid and silver doped bioactive glass nanoparticles. SEM images of the multilayer film before and after 7-day immersion on simulated body fluid solution are shown in Figure 5A. The resulting film supported L929 fibroblast cells adhesion and proliferation (Figure 5B), which were dependent on the particular order of the assembled layers. Silver ions released from the bioactive glass nanoparticles endowed the coating with antibacterial properties against both *E. coli* and *S. aureus*. These antibacterial properties were evaluated by the inhibition zones in the disk diffusion methodology (Figure 5C). Moreover, thanks to the hyaluronic acid-dopamine conjugate, the coating presented good adhesion strength assessed through lap-shear stress tests.

Gadkari et al. also reported the use of silver nanoparticle-loaded chitosan as an antibacterial agent in multilayers assembled on fabric [126]. Base layers of anionic poly(styrenesulfonate) and cationic poly(allyl hydrochloride) were assembled on cationized fabric to provide strong binding between fibres and main body layers of poly(styrenesulfonate) and chitosan or silver nanoparticles-loaded chitosan. PSS/CHI multilayers presented a considerable antibacterial effect against both *E. coli* and *S. aureus* due to a barrier mechanism/antifouling effect. Multilayers with silver nanoparticle-loaded chitosan were seen to be even more effective due to the gradual release of silver ions in addition to the barrier mechanism. The release of silver ions increased in going from 5 to 15 bilayers.

In another approach, Liu et al. immobilized bovine serum albumin capped Ag NPs on a nanoporous polysulfone substrate through Layer-by-Layer interfacial polymerization [127]. The thin film was developed by sequential assembly of m-phenylenediamine aqueous solution, trimesoyl chloride hexane solution and Ag NPs aqueous solution. Though the Ag NPs presented little effect on the film’s surface contact angle, roughness and charge, its incorporation has a large effect on antimicrobial activity against *E. coli*.

Meng et al. employed a 3-dimensional matrix of Layer-by-Layer assembled anionic poly(acrylic acid) (PAA) and cationic poly(dimethyldiallylammonium) (PDDA) polyelectrolytes for in situ growth of silver nanoparticles under UV irradiation [128]. The multilayer also served to protect the synthesized Ag NPs, prolonging the secretion of Ag ions for as long as 120 h. By varying the number of PAA/PDDA bilayers the released amount of silver ions and thus, the antibacterial efficacy against *E. coli* and *S. aureus* could be tuned.

Though silver nanoparticles are the most explored nanoparticle as antimicrobial agents, other metallic nanoparticles are also being studied. Kruk et al. developed polyelectrolyte-copper antimicrobial coatings on various surfaces such as silicon, quartz and gold [125]. Those authors sequentially adsorbed positively charged poly(diallyldimethylammonium) chloride and negatively charged copper nanoparticles. A SEM image of copper nanoparticle containing multilayer is shown in Figure 5D. The resulting film exhibited antibacterial action against *S. aureus* caused by cell lysis upon contact of the bacteria with the surface of the multilayer (Figure 5E).

## 8. LbL for Antiadhesive Surfaces

**Figure 6:**
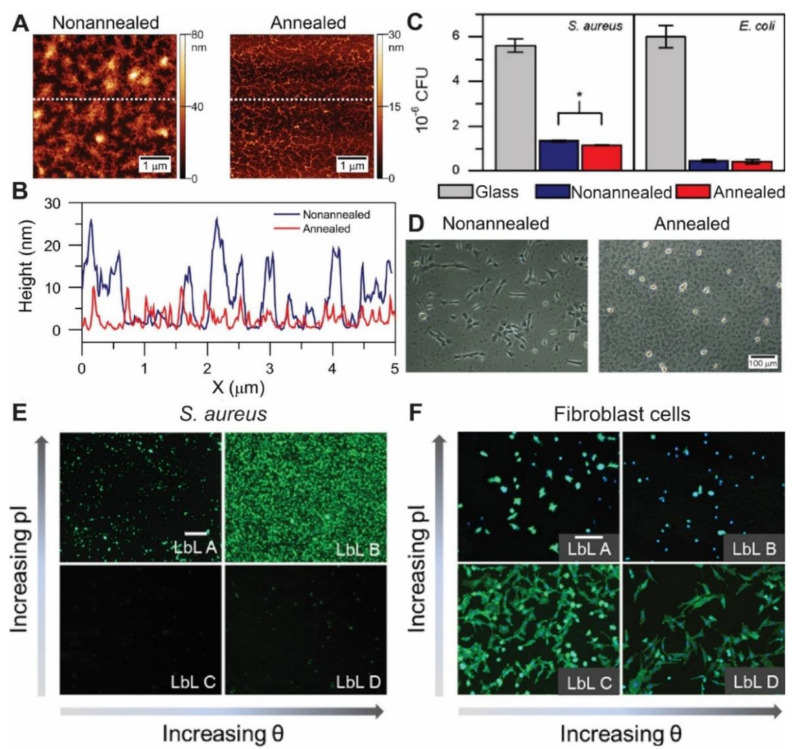
Strategies for assembling antiadhesive multilayer films. (**A**) AFM images of nonannealed and annealed (CHI/HA)_7_CHI multilayers. (**B**) AFM line profile from images depicted in (**A**) as indicated by the dashed line. (**C**) Colony-forming units (CFU) for *S. aureus* and *E. coli* bacteria detached from glass, nonannealed and annealed (CHI/HA)_7_CHI PEMs. (**D**) C2C12 cell images 24 h post seeding on nonannealed and annealed (CHI/HA)_7_CHI PEMs. (**E**) Live staining of adherent *S. aureus* on non-crosslinked LbL films with varying surface isoelectric point (pI) and water contact angle (θ). Scale bar is 100 µm. (**F**) Fluorescence microscopy images of adherent fibroblast cells stained with DAPI and phalloidin after 2 day seeding on non-crosslinked LbL films with varying surface isoelectric point (pI) and water contact angle (θ). Scale bar is 100 µm. (**A**,**B**) adapted with permission from [131], American Vacuum Society, 2017. (**C**,**D**) adapted with permission from [130], Elsevier, 2017. (**E**,**F**) adapted with permission from [132], American Chemical Society, 2018.

By means of combining chitosan (CHI), a positively-charged biopolymer with antimicrobial properties, and hyaluronan (HA), a nontoxic negatively-charged biopolymer it is possible to generate soft and highly hydrated films [129]. The CHI/HA multilayer assembly was optimized by changing the pH of the polymer which tunes the ionization degree of the polyelectrolytes, and by modifying the number of layers. Then, the adhesion and growth of *S. aureus* and *P. aeruginosa*, were studied on the LbL coated surfaces. The best results in preventing bacterial growth were observed when the surface density of primary amino groups of top CHI layer was maximized at pH 3 and following the assembly of 18 polyelectrolyte layers. Total suppression of *S. aureus*’s growth was observed, but little effect on *P. aeruginosa*, for which the authors propose the addition of antimicrobial agents. Using the same combination of polyelectrolytes but different assembly conditions (pH 5 and 15 layers), Muzzio et al. reported a 5-fold decrease in Gram-positive *S. aureus* and Gram-negative *E. coli* adhesion on film-coated surfaces with respect to control uncoated glass (Figure 6) [130]. Atomic Force Microscopy measurements of CHI/HA films assembled in these conditions revealed a fibrillar topography with 15–20 nm height and 200–300 nm width structures (Figure 6A,B). The adhesion was assessed by counting colony forming units and by GFP-expressing bacteria surface coverage quantification of fluorescent microscope images. The authors correlated the hydrophilicity of the CHI/HA films and their negative charge to the bacteria resistant characteristics, as both bacteria display a negative surface charge. The annealed films are slightly more negative and hydrophilic, and display a significant change in surface topography, with the treated multilayers being smoother (Figure 6A,B). Moreover, after thermal annealing of the films, a 72-h treatment at 37 °C, the adhesion of *S. aureus* decreased by a further 20% and no changes in *E. coli* adhesion were observed (Figure 6C). The authors correlated this bacteria-specific adhesion resistance mainly to these topographic differences, as *S. aureus* are not motile bacteria and possess no adaptable appendages to overcome the decrease in interaction area, while *E. coli* can adapt better to these topography changes as it has a complex machinery of flagella and fimbriae associated with the cellular wall. The scarce mammalian cell adhesion and protein absorption was even lower after thermal annealing, achieving a very antifouling coating as shown in Figure 6D [39,131].

In a recent study, Guo et al. studied changes in physicochemical properties of the multilayer prepared with the LbL technique such as wettability, surface charge and stiffness affected the tissue bacteria/surface and cell/surface interactions (Figure 6E,F) [132]. To this end, they employed branched polyethylenimine (PEI) and custom-synthesized polyanions with either poly-(ethylene glycol)- or alkyl-carboxylic side-chains. After assembly, they cross-linked the multilayers to produce stiffer films. *S. aureus* and *E. coli* adhesion was controlled only by the charge and wettability of the surface and was independent of mechanical properties of the film. A negative charge and hydrophilic surface hindered both bacteria adhesions (Figure 6E) [132]. On the other hand, while the adhesion of 3T3 fibroblasts was strongly dependent on surface charge and wettability for soft films, a good cell adhesion was always observed on cross-linked, and thus stiffer films, which was not dependent on surface charge and wettability (Figure 6F). One can conclude from this work that by fine tuning the physicochemical properties of PEMs it is possible to selectively control/promote or prevent the adhesion of mammalian cells and bacteria.

Lu et al. [133] developed a Layer-by-Layer bacteria-responsive hydrogel with pH-triggered hydrophobicity. They employed as building blocks polyvinylpyrrolidone (PVPON) and different polyacids with increasing alkyl side chain length: commercial polymethacrylic acid (PMAA) and poly(2-ethylacrylic acid) (PEAA), poly(2-n-propylacrylic acid) (PPAA) or poly(2-n-butylacrylic acid) (PBAA). These multilayers showed an increase in elastic modulus and a decrease in water uptake with increasing polyacid hydrophobicity, i.e., with increasing length of the alkyl side chain. At physiologic pH 7.4, the more hydrophobic hydrogels showed a superior resistance to *S. epidermidis* colonization. As a result of bacteria proliferation and metabolism, the pH of the medium decreases. As the media acidifies the more hydrophobic hydrogels tend to dehydrate and kill bacteria upon contact by exposing polyvinylpyrrolidone cationic groups, which protonate at acidic pHs, and are capable of disrupting bacteria membranes. Both the antiadhesive and killing efficiency were enhanced by the polymer hydrophobicity. These hydrogels do not display free cationic groups under physiological conditions, and displayed good adhesion and cytocompatibility for human osteoblasts.

Polyelectrolyte multilayers can also be prepared by host–guest interaction. Taking into account the results reported on the antifouling properties of PEI and the antimicrobial properties of chitosan (CHI) [134], the authors assembled a multilayer of synthetic polyethylenimine-β-cyclodextrin (PEI-β-CD) and synthetic ferrocene-modified chitosan (Fc-CHT). The cyclodextrin and ferrocene modifications allow for the build-up of the film applying the Layer-by-Layer principle of sequential assembly through coordination chemistry of two polymers of the same charge. The antifouling and antimicrobial performance against *Pseudomonas sp.* and *S. aureus* increased with the number of host-guest bilayers.

## 9. Multifunctional Antimicrobial Multilayers

Each antibacterial strategy, such as loading of nanoparticles, antimicrobial peptides and antibiotics or the engineering of the multilayer to endow it with contact-killing or antiadhesive properties, has its advantages and limitations. The versatility of the LbL assembly technique lies in the possibility of the synergistic integration of different strategies in order to generate multifunctional coatings.

In this vein, Tang et al. loaded polyelectrolyte multilayers (PEMs) composed of poly(allylamine hydrochloride) (PAH) and poly(acrylic acid) (PAA) with silver nanoparticles (Ag NPs) [135]. The hydrated, swollen nature of the PAH/PAA multilayers resulted in an anti-adhesive coating, and the immobilization of the Ag NPs within the film enhanced the contact probability of NPs and bacteria, increasing the killing efficiency.

Enzymes that generate antimicrobial agents such as hydrogen peroxide can be incorporated in multilayers films to confer it with bactericidal properties. In reference [136] the authors immobilized the antibacterial hydrogen peroxide (H_2_O_2_) producing enzyme cellobiose dehydrogenase (CDH) in a multilayer with novel antifouling and contact killing polymeric building blocks. They synthetized an antifouling copolymer with zwitterionic and quaternary ammonium side groups and a contact killing derivative of that polymer with octyl groups. The resulting polycations were alternatively assembled with poly(styrenesulfonate) as the polyanion. The enzyme CDH was embedded as a polyanionic layer in the multilayer system due to its negative charge. The location of the enzyme in the multilayer was found to influence its activity. The resulting films presented both antifouling and antimicrobial properties [136].

Kurapati et al. [137] synergistically combined two kinds of antimicrobial therapies using the Layer-by-Layer assembly technique. The authors assembled 20 to 80 layers of positively charged poly(allylamine hydrochloride) and negatively charged graphene oxide, which has intrinsic antimicrobial properties by damaging the bacteria cell wall with its sharps edges (nanoknives). The increase in the number of layers leads to an increase in antibacterial activity against *E. coli* due to the increase in film roughness and graphene oxide content. By exposing the films to a near-infrared lasers the authors exploited the photothermal properties of graphene oxide and enhanced the coating antibacterial efficiency. The results suggest that the combined antimicrobial effects (membrane stress + photothermal heating) cause increased bacteria lysis in comparison to the individual effects [137]. A brief summary of the LbL assemblies for antibiotic encapsulation and with other antibacterial mechanisms are presented in Table 1.

## 10. Layer-by-Layer Assembly among Other Localized Antibacterial Strategies

The use of Layer-by-Layer coating is one approach among the many explored for the development of antimicrobial coatings that can be found in literature [6,41,138]. Polymer grafting is an attractive approach, which offers similar advantages to LbL regarding encapsulation of antibiotics, functionalization with peptides, and used in combination with other antibacterial materials [139]. For example, ionic polymers such as poly(sodium styrene sulfonate (polyNaSS) have been grafted to a titanium surface, reducing the adhesion of *S. aureus*, and which can be further used to immobilize antimicrobial molecules [139,140]. Covalent bonding offers a strong link between the coating and the material, resulting in a durable interface [141], but the chemical reactions needed to covalently bind the polymers often result in sparse films. Additionally there are difficulties to covalently attach a polymer over a relatively large surface in an implant [142]. LbL on the other hand is easy to apply over large areas by either dipping or spraying.

Surface nanostructuring has also gained attention as a means to reduce bacteria proliferation. Ercan et al. [143] showed that surface modification by depositing Ti nanotubes changes the bacterial response. Other works have also demonstrated the effectivity against *S. aureus*, *S. epidermidis* and *P. aeruginosa* [143,144], This approach is based on the development of anti-adhesion surfaces; thus, they are not only effective in preventing bacterial adhesion but they also inhibit the adhesion of osteoblasts. Due to the weak adhesion of osteoblasts, the integration of the implant into the surrounding tissue cannot occur [145]. As we have also seen, LBL can be used to decrease bacteria adherence to surfaces in conditions where osteoblast adherence is also hindered.

Another potential strategy for a localized antibacterial action is based on incorporating ions with a bactericidal action on the surface of the implant. Ion-implanted surfaces with elements such as fluorine (F), zinc (Zn) calcium (Ca), chlorine (Cl), iodine (I), copper (Cu), cerium (Ce) or selenium (Se), all displaying an antibacterial action can be incorporated to the coating through anodic oxidation of the ions. By the Magnetron Sputtering (MS) technique, a plasma is made to interact with a solid target in a vacuum reactor, producing the sputtering of atomic species from a well-defined race-track region, and their deposition on a substrate located a few centimetres away [146]. Several works have used this technique to implant ions in the Ti coating [25,147,148,149]. The hydroxylation of this ions once they are in contact with bacteria leads to the formation of reactive components (HCl, HOCl or H_2_O_2_, to name but a few), which cause oxidation of the cell membranes, leading to an increased permeability of cells, and finally to cell death [150]. Consequently, these reactive compounds are also going to interfere with osteoblast proliferation, avoiding implant proper integration.

## 11. Concluding Remarks and Future Perspectives

The lLayer-by-Layer technique offers multiple possibilities for the design of antibacterial coatings that can be assembled on implants. Some of the approaches revised in this manuscript have been directed towards antibacterial coatings in different scenarios from a bone implant, but could also be adapted for coating an implant. The simple, straightforward and inexpensive methodology behind LbL: a sequential assembly of oppositely charged polyelectrolytes from aqueous solutions, and the possibility of spraying polyelectrolyte for coating larger surfaces makes LbL highly appealing for implant coating in comparison with other potential polymeric coating methods, like polymer brushes or hydrogels when we think of covering large surfaces. LbL assemblies can be used to load antibiotics on the implant surface for a localized and prolonged release at the site of the implant. LbL can also be used to decorate the implant with antibacterial peptides and nanoparticles, and by choosing the proper polymers and assembly conditions LbL films can prevent bacteria adhesion on the surface. Moreover, LbL can combine all strategies in a single film, enhancing the bactericidal potential of the coating and allowing for the design of strategies for fighting biofilm formation and bacterial infections, while at the same time the formation of tissue on an implant is not harmed. This is indeed the main goal of LbL research for antibacterial coatings should target now. PEMs must be designed as antibacterial and osteogenic. Issues such as the dosage of encapsulated antibiotics must be also addressed. An optimal coating with encapsulated antibiotics will demand a larger release during surgery and immediately after since the risk of infection is high. At the same time a continuous antibacterial action but with a smaller dose of antibiotics being released for weeks is necessary to avoid the occurrence of bacterial focus. LbL films could be used for encapsulating more than one antibiotic to be delivered in a synergistic or sequential manner. Another challenge for LbL films is the design of coatings that in the case of formation of a biofilm could be capable of fighting it.

Safety issues also represent a challenge for LbL coatings. While having an antibacterial action, the coating should not pose a risk to humans. It should also not lead to cell death or inflammatory responses, and should not hinder cells from adhering to the implant. Many of the examples provided in this review involve components, polymers or nanomaterials, of synthetic origin, that are not necessarily biodegradable, which may represent some risk for the host. Most likely, LbL films based on biodegradable polyelectrolyte have more potential for translation. However, “the dose makes the poison”, and the amount of polymer per layer of polyelectrolyte is in the range of a few to tens of ng per cm^2^ [151], which may translate in exposing the patients to very small doses of nanomaterials or polymers. Despite the number of publications on the topic of LbL for antibacterial applications few works have reached in vivo experimentation, which is a necessary step for future applications and for a proper safety evaluation of the coatings.

## Figures and Tables

**Table 1:** Summary of antibacterial Layer-by-Layer strategies reviewed in this article.

Antibacterial Mechanism	Composition	Bacteria Tested	Findings	Reference
Antibiotic Encapsulation	Gentamicin sulphate, PAA, Poly 1	*S. aureus* (Gram+)	Bactericidal effect and nontoxic towards MC3T3-E1 pre-osteoblasts	[11]
Antibiotic Encapsulation	Poly(methacrylic acid), SNARF-1, gentamicin or polymyxin B	*S. aureus* (Gram+) and *E. coli* (Gram−)	Antibiotic release is triggered by pH changes. High bactericidal efficiency	[99]
Antibiotic Encapsulation	PAA, PLL and tetracycline	*Porphyromonas gingivalis* (Gram−)	Initial burst release of tetracycline, which antibacterial effects	[100]
Antibiotic Encapsulation	PAA-Gentamicin complexes and PLL	*S. aureus* (Gram+)	Prevents bacteria proliferation with low number of bilayers. Antibiotic burst release followed by a prolonged release	[101]
Antibiotic Encapsulation	Vancomycin-loaded niosomes and PLA	*S. aureus* (Gram+)	Antibacterial activity with no cytotoxic effects on L929 mouse fibroblast cells	[102]
Antibiotic Encapsulation	Gelatin, BMP-2, CHI, Vancomycin	*S. aureus* (Gram+)	Bactericidal effect and enhanced osteogenic differentiation of mesenchymal stem cells	[105]
Antimicrobial Peptides Encapsulation	Polydopamine, ε-polylysine, gum Arabic	*S. aureus* (Gram+) and *E. coli* (Gram−)	Long-term antibacterial properties and improved proliferation and osteogenic differentiation of rBMSCs	[115]
Antimicrobial Peptides Encapsulation	Gelatin, CHI, (Pac-525)-loaded PLGA microspheres	*S. aureus* (Gram+) and *E. coli* (Gram−)	Bactericidal activity for one week and bacteriostatic activity for up to a month. Good adhesion, proliferation and osteogenic differentiation of rBMSCs	[116]
Antimicrobial Peptides Encapsulation	Polyphenolic tannic acid and parasin I	*S. aureus, S. epidermidis* (Gram+) and *E. coli, Pseudomonas sp.* (Gram−)	The coating exhibits good resistance to bacteria adhesion	[118]
Antimicrobial Peptides Encapsulation	CHI, HA and Tet213 linked to collagen IV	*S. aureus* (Gram+) and *P. gingivalis* (Gram−)	Bacteria growth inhibition for up to a month. Multilayer presents good adhesion of keratinocyte cell line and non-cytotoxicity	[119]
Antibacterial Nanoparticles Loading	CHI, dopamine-modified HA, silver doped bioactive glass NPs	*S. aureus* (Gram+) and *E. coli* (Gram−)	Antibacterial properties against both bacteria. The film supports L929 fibroblast cells adhesion and proliferation. Good adhesion strength to other materials.	[124]
Antibacterial Nanoparticles Loading	PSS, PAH, silver nanoparticle-loaded chitosan	*S. aureus* (Gram+) and *E. coli* (Gram−)	Antibacterial effect due to due to a barrier mechanism/antifouling effect and silver ions release	[126]
Antibacterial Nanoparticles Loading	BSA capped Ag NPs, m-phenylenediamine, trimesoyl chloride	*E. coli* (Gram−)	Film assembled through Layer-by-Layer interfacial polymerization with good antimicrobial activity	[127]
Antibacterial Nanoparticles Loading	PAA, PDDA, in situ growth of silver NPs	*S. aureus* (Gram+) and *E. coli* (Gram−)	Antibacterial efficacy tuned by varying the number of PAA/PDDA bilayers	[128]
Antibacterial Nanoparticles Loading	PDDA and copper NPs	*S. aureus* (Gram+)	Antibacterial action caused by cell lysis upon contact of the bacteria with the surface of the multilayer	[125]
Antiadhesive Surface	CHI, HA	*S. aureus* (Gram+) and *P. aeruginosa* (Gram−)	Antibacterial properties can be tuned by changing the pH of the polymer solutions and number of layers. Total suppression of *S. aureus*’s growth, little effect on *P. aeruginosa*.	[129]
Antiadhesive Surface	CHI, HA	*S. aureus* (Gram+) and *E. coli* (Gram−)	Low bacteria adhesion on films. After thermal annealing of the films, *S. aureus’* adhesion further decreases	[130]
Antiadhesive Surface	Branched PEI and custom-synthesized polyanions	*S. aureus* (Gram+) and *E. coli* (Gram−)	Surface charge and wettability control bacteria and 3T3 fibroblasts adhesion on non-cross-linked soft films	[132]
Antiadhesive Surface	PVPON and different polyacids with increasing alkyl side chain length	*S. epidermidis* (Gram+)	Film with pH-triggered hydrophobicity with antiadhesive and bactericidal properties.	[133]
Antiadhesive Surface	PEI-β-cyclodextrin and synthetic ferrocene-modified CHI	*S. aureus* (Gram+) and *Pseudomonas sp.* (Gram−)	Antifouling and antimicrobial performance increase with the number of bilayers	[134]
Multifunctional Antibacterial Multilayer	PAH, PAA, silver NPs	*E. coli* (Gram−)	Antiadhesive properties due to PAH/PAA and bactericidal properties due to silver NPs	[135]
Multifunctional Antibacterial Multilayer	Cellobiose dehydrogenase, synthetic antifouling copolymer and PSS	*S. aureus* (Gram+)	The location of the hydrogen peroxide-producing enzyme in the multilayer was found to influence its activity. The films presented both antifouling and antimicrobial properties	[136]
Multifunctional Antibacterial Multilayer	PAH and graphene oxide	*E. coli* (Gram−)	Combined antimicrobial effects (membrane stress + photothermal heating) cause increased bacteria lysis in comparison to the individual effects	[137]

**Abbreviations**: poly acrylic acid (PAA), poly(β-amino ester) (Poly 1), Gram positive (Gram+), Gram negative (Gram−), poly-L-lysine (PLL), poly(lactic acid) (PLA), bone morphogenetic protein-2 (BMP-2), chitosan (CHI), poly lactic-co-glycolic acid (PLGA), rat bone marrow mesenchymal stem cells (rBMSCs), hyaluronic acid (HA), nanoparticles (NPs), poly(styrenesulfonate) (PSS), poly(allyl hydrochloride) (PAH), Bovine serum albumin (BSA), poly(dimethyldiallylammonium) (PDDA), polyethylenimine (PEI), polyvinylpyrrolidone (PVPON).

## Data Availability

Not applicable.
